# Improvement of optimum pH and specific activity of pectate lyase from *Bacillus* RN.1 using loop replacement

**DOI:** 10.3389/fbioe.2023.1242123

**Published:** 2023-07-04

**Authors:** Piwu Li, Xiaofeng Wei, Yun Wang, Hui Liu, Yanpeng Xu, Ziyang Zhang, Junlin Li, Jianbin Wang, Chuanzhuang Guo, Songsen Sui, Junqing Wang, Ruiming Wang

**Affiliations:** ^1^ State Key Laboratory of Biobased Material and Green Papermaking (LBMP), Qilu University of Technology, Jinan, Shandong, China; ^2^ Department of Biological Engineering, Qilu University of Technology, Jinan, Shandong, China; ^3^ Zhucheng Dongxiao Biotechnology Co. Ltd., Zhucheng, Shandong, China

**Keywords:** pectate lyases, alkali resistance, loop replacement, enzyme activity, molecular dynamics simulation

## Abstract

**Background:** Alkaline pectate lyase plays an important role in papermaking, biological refining and wastewater treatment, but its industrial applications are largely limited owing to its low activity and poor alkali resistance.

**Methods:** The alkaline pectate lyase BspPel from *Bacillus* RN.1 was heterologously expressed in *Escherichia coli* BL21 (DE3) and its activity and alkali resistance were improved by loop replacement. Simultaneously, the effect of R260 on enzyme alkaline tolerance was also explored.

**Results:** Recombinant pectate lyase (BspPel-th) showed the highest activity at 60°C and pH 11.0, and showed significant stability over a wide pH range (3.0–11.0). The specific enzyme activity after purification was 139.4 U/mg, which was 4.4 times higher than that of the wild-type enzyme. BspPel-th has good affinity for apple pectin, since the *V*_max_ and *K*_*m*_ were 29 μmol/min. mL and 0.46 mol/L, respectively. Molecular dynamics simulation results showed that the flexibility of the loop region of BspPel-th was improved.

**Conclusion:** The modified BspPel-th has considerable potential for industrial applications with high pH processes.

## 1 Introduction

Pectin exists in the intercellular layer and cell wall of plant cells and is composed of α-1,4-galacturonosidic linkages connected to galacturons. It is a type of heteropolysaccharide with weak acidity and strong heat resistance, mainly composed of galacturonic acid, rhamnose, galactose, and arabinose (Yadav et al., 2009; Amin et al., 2019). Pectin participates in the cross-linking of cellulose, hemicellulose and lignin rendering the plant tissue structure firm and hindering the invasion of pathogenic microorganisms (Ahmed et al., 2021).

Pectinase is the general name for a series of enzymes that can catalyze the degradation of pectin substances, which can be divided into protopectinase, pectinesterase and pectin depolymerase according to the mode of action (Amin et al., 2019). Pectate lyases (EC 4.2.2.2) are pectin depolymerization enzymes that cleave α-1,4-galacturonosidic linkages of polygalacturonic acid (PGA) and via β-elimination, remove one H atom at the C5 position of galacturonic acid to form unsaturated double bonds. At present, pectate lyase strains have been reported, alkaline pectate lyase genes from *Erwinia* (Jenkins et al., 2004), *Pseudomonas* (Sharma et al., 2011) and *Bacillus* (Nasser et al., 1990; Mei et al., 2013; Zou et al., 2013) have been expressed through gene engineering technology.

The molecular weight of pectate lyase is generally between 20 and 60 kDa, its optimum temperature is 50°C–70°C, it exhibits good activity under alkaline conditions (pH 8–10). pectate lyases from fungi are generally be inactivated above 50°C, but the pectate lyase from *Bacillus* has a higher tolerance to temperature. The optimum pH of Pel4-N, Bsp165PelA and pel4J4 were 11.5, which are the most alkali-resistant pectinase found in this study (Wu P. et al., 2020). Pectate lyase is widely used in papermaking (Amin et al., 2019), biological refining of cotton fiber (Abdel-Halim et al., 2010; Wang et al., 2015; Zhou et al., 2017b; Cheng et al., 2019; Zhou and Wang, 2021), degumming of plant phloem fiber (Zhou et al., 2017a; Zhen et al., 2020), treatment of pectin-containing wastewater, and in other fields necessitating the degradation of pectin (Kohli and Gupta, 2015). The industrial use of enzymes can contribute to solving environmental and energy problems associated with traditional processes, in this respect, pectate lyase is an enzyme of notable commercial value (Parthasarathy and Murthy, 2000; Radestock and Gohlke, 2011; Raksha et al., 2023). Presently, owing to the low activity and poor stability of enzymes, most of them are limited to research and laboratory applications. BspPel has previously been expressed and characterized (Zheng et al., 2020), but its industrial application is also currently limited on account of by its low enzymatic activity and poor alkali resistance. In order to circumvent the abovementioned limitations, there is necessary to produce enzymes with high activity and resistance to alkali.

In this study, we significantly improved the alkali resistance and enzymatic activity of BspPel by loop replacement from *Bacillus* RN.1 heterologously expressed in *Escherichia coli* BL21 (DE3), its the highest activity showed at 60°C and pH 11.0, and showed significant stability over a wide pH range (3.0–11.0). The specific enzyme activity was 4.4 times higher than that of the wild-type enzyme, reached 139.4 U/mg after purification. The modified BspPel-th has considerable potential for industrial applications, and the strategies described here also can be implemented for the engineering of other industrial enzymes in order to enhance their characteristics.

## 2 Materials and methods

### 2.1 Bacterial strain plasmids and materials

*E. coli* BL21 (DE3) cells were purchased from Shanghai Bioengineering (St. Louis, MO, United States). The strain information of *Bacillus* sp. RN1, isolated from a hot spring in Ranong Province, Thailand (Sukhumsiirchart et al., 2009), was searched from NCBI database (https://www.ncbi.nlm.nih.gov/). The plasmids pET28a (+)-*pel*, pET28a (+)-*pel-th* and pET28a (+)-*pel-th*/R260S were constructed in our laboratory. PGA, apple pectin and citrus pectin were purchased from Sigma-Aldrich (St. Louis, MO, United States). All chemicals were of analytical grade and obtained from commercial suppliers.

### 2.2 Gene cloning and expression plasmid construction

The NCBI BLAST program was used to search for the nucleotide sequence of *BspPel*. The SignalP 5.0 server (https://services.healthtech.dtu.dk/service.php?SignalP-5.0) was used to predict the signal peptide, which was removed. The *BspPel* gene (No. BAG12908) from *Bacillus* RN.1 was optimized and amplified (GenScript Biotechnology Co., Ltd., China) obtain the gene pel (accession no. OQ871586), with *Nco*I and *Xho*I restriction sites to clone *pel* into pET28a (+) to construct the pET28a (+)-*pel* plasmid. Pel4-N (No. AB042100) from the alkaliphilic *Bacillus* sp. strain P-4-N has a specific enzyme activity of 2.38 U/mg in culture supernatants; and given its excellent optimal pH 11.5 (Kobayashi et al., 2000), we selected a 12-amino acid sequence of Pel4-N (identified after sequence alignment) for fragment replacement.

To amplify pET28a (+)-*pel-th*, we used the primer pair th-F (5′- GGA​TTC​TGG​CAT​AGA​GAG​TCG​AAC​AGG​TTA​CTG​GCA​TGT​TT-3′) and th-R (5′-ATG​CCC​AGA​TCC​ACG​GGG​TCA​TTT​CGG​AAC​AGA-3′), and the following amplification program: an initial denaturation at 95°C for 3 min; followed by 30 cycles at 95°C for 15 s, 60°C for 15 s, and 72°C for 30 min; and a final extension at 72°C for 5 min. DNA sequencing confirmed that the obtained gene was a recombinant pectate lyase gene, and the plasmids were transformed into *E. coli* BL21 (DE3) for expression. To examine the effects of amino acids on alkali tolerance, we performed a site-specific mutation of the arginine at position 260 of BspPel-th using the site-directed mutation primers: pel-th/R260S-F (5′-TAG​CAG​Tag​cAC​AGG​TTA​CTG​GCA​TGT​TTC​CAA-3′) and pel-th/R260S-R (5′-AAC​CTG​Tgc​tAC​TGC​TAT​GCC​AGA​ATC​CTA​ACG-3′). The plasmid pET28a (+)-*pel-th/R260S* was obtained by amplification under the aforementioned PCR conditions and was also transformed into *E. coli* BL21 (DE3) for expression analysis.

### 2.3 Expression and purification of BspPel-th

In order to overexpress pectate lyase, the constructed strain *E. coil* BL21 pET28a (+)-*pel-th* was inoculated into 50 mL of Luria-Bertani (LB) liquid medium supplemented with 50 μg/mL kanamycin, at an inoculum concentration of 1%, and incubation temperature of 37°C. When the OD_600_ was between 0.7 and 0.8, we added 0.5 mmol/L IPTG to the culture and expression was induced at 25°C, with shaking at 200 rpm for 12 h. For purification, 6× His-labeled proteins were centrifuged for 15 min at 12,000 × g for at 4°C to obtain cells, which were then sonicated on an ice bath phosphate buffer (0.2 M, pH 7.4) for 20 min. The cell disruption procedure is: 4 s of broken, 6 s of stay, power 300 W. The cell fragments were removed by centrifuging for 15 min at 12,000 × g and 4°C. The protein supernatant was loaded onto a pre-equilibrated Ni-NTA agarose gel column, and the column was washed with a washing buffer (20 mM Tris, 250 mM NaCl, 20 mM imidazole, pH 7.4). The bound proteins were eluted using elution buffer (20 mM Tris, 250 mM NaCl, and 200 mM imidazole, pH 7.4). Protein concentrations were measured using the Bradford method, and protein purity was analyzed by sodium dodecyl sulphate-polyacrylamide gel electrophoresis (SDS-PAGE).

### 2.4 BspPel-th activity assay

Pectate lyase activity was determined by measuring the increase in unsaturated bonds generated at 235 nm. Enzyme activity assays were performed as described by Li et al. (2022) with some modifications. The reaction mixture consisted of 1900 μL 50 mM Gly-NaOH (pH 11) containing 0.2% substrate and 100 μL appropriately diluted enzyme solution. The reaction was carried out at 60°C for 10 min, and then terminated by adding 3 mL of 30 mM H_3_PO_4_. Absorbance was measured spectrophotometrically at 235 nm (Shanghai Youke Instrument Co., Ltd., Shanghai, China) to determine the formation of unsaturated products. A unit of pectate lyase activity was defined as the production of 1 μmol of unsaturated oligo-gacturonic acid per min. The molar extinction coefficient of the enzyme amount of the unsaturated bond was 4600 M^−1^cm^−1^. All activity measurements were repeated three times.

### 2.5 Effects of temperature, pH and metal ions on enzyme activity and stability

The optimum temperature for enzyme activity was determined by carrying out reactions at temperatures ranging from 45°C to 85°C for 10 min in 50 mM Gly-NaOH (pH 10), a standard reaction buffer, containing 0.2% pectin (Sheladiya et al., 2022). The optimum pH was determined at 60°C in 50 mM glycine NaOH buffer (pH 8.5–12) containing 0.2% pectin. The residual enzyme activity was measured after incubation at 40°C, 50°C, 60°C and 70°C, and the enzyme activity before incubation was taken as the initial enzyme activity. At 25°C, the pH stability of the enzyme was determined by incubation in 50 mM disodium hydrogen phosphate citric acid (pH 4.0–7.0), 50 mM Tris-HCl (pH 8.0), and 50 mM Gly-NaOH (pH 9.0–12.0) buffer solution for 6 h.

In the reaction system, the effects of Ca2+, Mg2+, Ni2+, Mn2+, Cu2+, K+, Na+, Zn2+, Fe2+, Fe3+, ethylenediamine tetraacetic acid (EDTA), and SDS on enzyme activity were evaluated at final concentration of 0.05 mM or 0.1% (Zheng et al., 2020). Under standard reaction conditions, the degree of inhibition or activation of enzyme activity was measured and the percentage of enzyme activity in the reaction system without the addition of metal ions or chemical reagents was measured.

### 2.6 Substrate specificity and kinetic parameters

Gly-NaOH buffer (50 mM) was used to prepare different substrates at a concentration of 0.2% (w/v), and the activity was measured to determine the substrate specificity, including PGA, apple pectin, and citrus pectin. The A235 method was used to determine the kinetic parameters of BspPel and BspPel-th on pectin, with a concentration range of 0.1–8 mg/mL (Sheladiya et al., 2022). The reaction was performed under standard conditions for 10 min. Plots derived from data using non-linear fitting methods were constructed.

### 2.7 Three-dimensional modeling and molecular dynamic simulation

The three-dimensional structure of BspPel and BspPel-th proteins was determined using SWISS-MODEL (https://swissmodel.expasy.org/) online web page prediction. PyMOL software was used to display the three-dimensional structure. Molecular dynamics (MD) simulation was used to study the protein flexibility of BspPel and BspPel-th, providing a basis for the improvement of mutant enzyme activity at the level of overall protein structure and replacement fragments. MD simulation was carried out using GROMACS 4.5 software (http://www.gromacs.org/). The box shape was a cube, and the size of the boundary atoms in all directions extended outward by 1.5 nm. After the box was filled with water molecules, ions were added to make the entire system electrically neutral, and then the energy was minimized (Li, et al., 2022). The system was simulated for 40 ns The atomic displacement parameter (B-factor) of amino acid residues was simulated and calculated using B-FITTER.

## 3 Results

### 3.1 Gene cloning and sequence analysis of BspPel-th

Signal peptide prediction identified the first 27 AAs from *Bacillus* RN.1. Recombinant BspPel synthesized after removing the signal peptide was used as a template. Pel4-N which has high alkali resistance, encodes 333 amino acids. The amino acid sequence identical to those of Pel4-N and BspPel was 65% by homology comparison. The 250–261 amino acids of BspPel were replaced by residues 268–279 of Pel4-N to obtain the recombinant pectin lyase BspPel-th. These 12 amino acids are located in the loop region of the three-dimensional structure of the protein.

BspPel-th showed 95.9% similarity to the protein sequence from *Bacillus licheniformis* S91 (GenBank ANS59493.1), whereas identities of 83%, 68%, 66%, 65%, and 65% were obtained for the PelB from *Bacillus* (GenBank no. WP_046129374.1), *Bacteroid* 598k (WP_128659822.1), KSM-K16 (BAD62606.1), BacPelA (ALK03050.1), and *Bacillus* Pel4-N (BAA96478.1), respectively, based on amino acid sequence homology. BspPel-th was identified as a member of the PL1 family.

**FIGURE 1:**
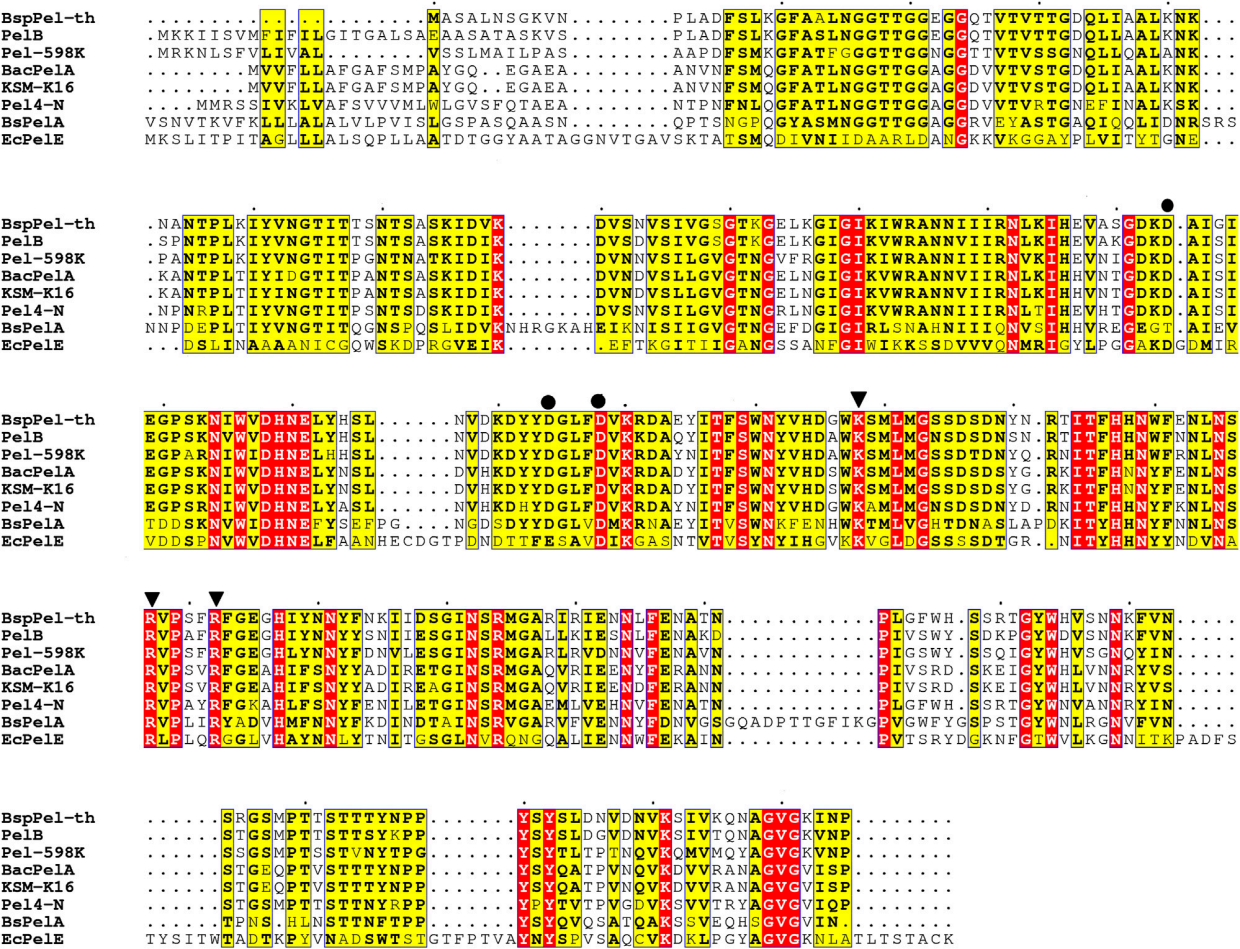
Sequence alignment of pectate lyases. PelB from *Bacillus* sp. (accession no. WP_046129374.1), *Paenibacillus* sp 598 K (WP_128659822.1), BacPelA from *B. clausii S10* (ALK03050.1), KSM-K16 (BAD62606.1) and Pel4-N from *Bacillus* sp. P-4-N (BAA96478.1), BsPelA (ACY38198.1), EcPelE from *E. chrysanthemi* (WP 039999250.1).

Presently, nine different crystal structures of pels have been reported in the PL1 family (http://www.cazy.org/PL1_structure.html). The details regarding the structurally characterized Pels suggest that BspPel-th showed high similarity to BsPelA from *Bacillus* sp. N16-5 and EcPelE from *Erwinia chrysanthemi* with 43% and 27% identity, respectively. Therefore, these two sequences were used for multiple protein sequence alignments (Figure 1). K178 and R207/R212 of BspPel-th are suggested to play a comparable role as the essential catalytic bases and D124, D154, and D158 are completely conserved in BspPel-th for Ca^2+^ interaction (Lietzke et al., 1996; Zheng et al., 2012; Tu et al., 2017).

### 3.2 Expression and purification of recombinant enzyme

**FIGURE 2:**
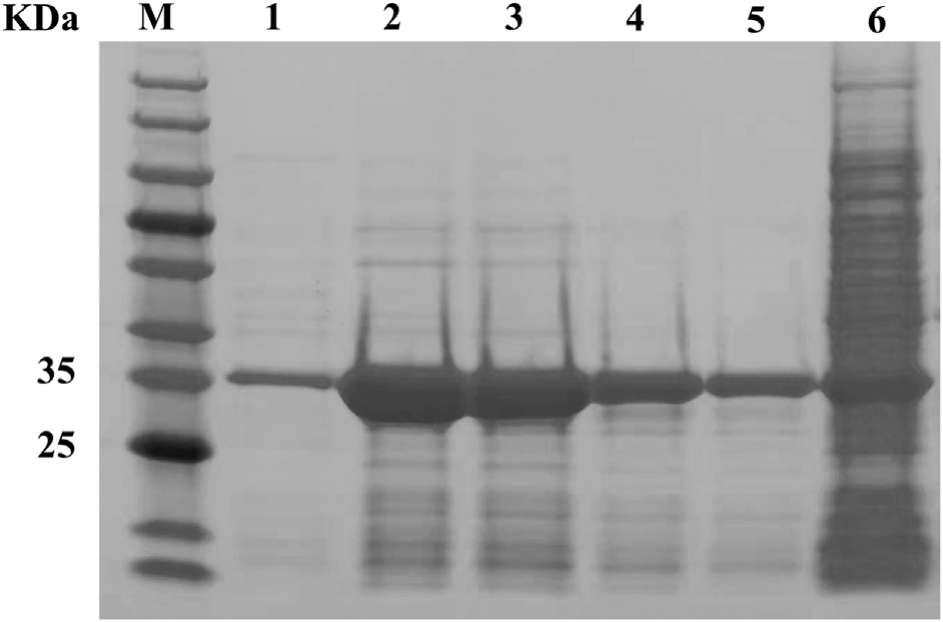
SDS-PAGE analysis of BspPel-th. Lane M, molecular weight marker; Lane 1–5, purified enzyme; lane 6, supernatant of crude extract from *E. coli* BL21 (DE3) harboring pET28a-*pel-th*.

The pectate lyase activity was detected under standard conditions. After 12 h induction by 50 mL of medium, crude enzyme activity reached 26.2 U/mL and after purification, the specific enzyme activity 139.4 U/mg. SDS-PAGE analysis showed that the molecular weight of the purified recombinant mature BspPel-th was approximately 35.0 kDa, which was consistent with the predicted results (Figure 2). The crude enzyme activity of BspPel under the same induction conditions was 8.9 U/mL, and the specific enzyme activity was 31.6 U/mg. The specific enzyme activity of the mutant after recombination was 4.4 times higher than that of the wild type.

### 3.3 Biochemical properties of BspPel-th

**FIGURE 3:**
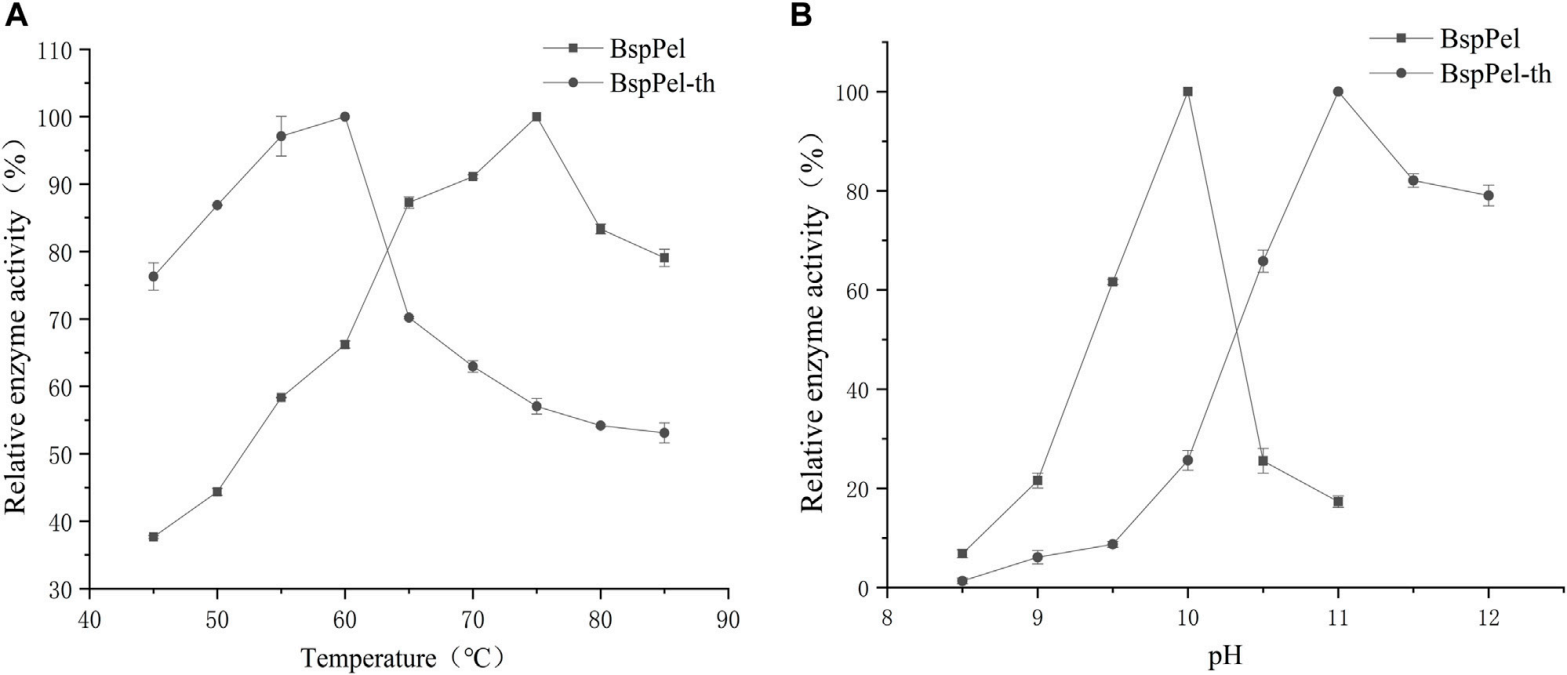
Temperature and pH distribution of BspPel and BspPel-th. **(A)** optimum temperature, **(B)** optimum pH. Values are expressed as the mean of three experiments. Error bars represent standard deviations.

Two factors that affect the enzyme activity are temperature and pH. The alkaline pectin lyases of *Bacillus* have been reported to have optimal activity at temperatures of between 40°C and 70°C and pH values ranging from 8.0 to 10.5 (Purmonen et al., 2007; Li et al., 2010; Zhang et al., 2014; Liang et al., 2015). The activities of BspPel and BspPel-th were detected using pectin as a substrate (Figures 3A, B). It can be seen that the optimum temperatures of BspPel is 75°C, and the optimum pH is 10. The reconstituted BspPel-th retained more than 60% activity over the temperature range of 45°C–70°C. The optimum temperature was 60°C, and the optimum pH was increased from 10 to 11.

**FIGURE 4:**
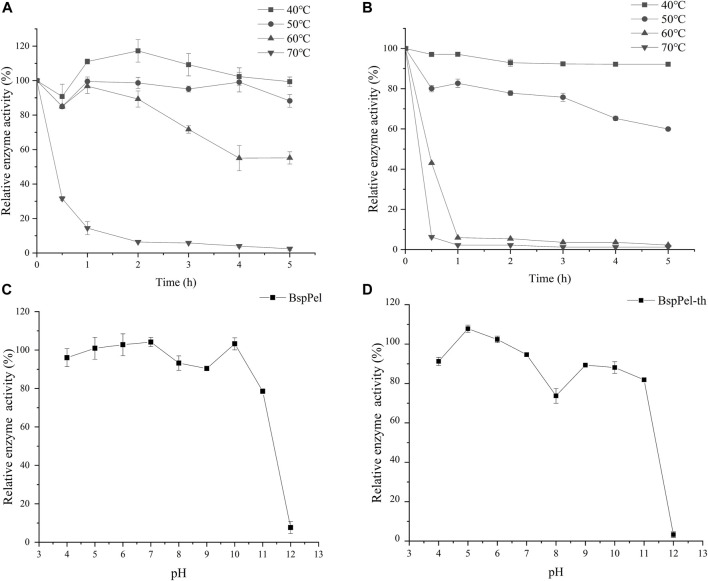
Temperature and pH distribution of BspPel and BspPel-th. **(A)** BspPel temperature stability, **(B)** BspPel-th temperature stability, **(C)** BspPel pH stability, **(D)** BspPel-th pH stability. Values are expressed as the mean of three experiments. Error bars represent standard deviations.

The thermal stability assays showed that BspPel-th still retains more than 60% activity after incubating at 40°C–50°C for 5 h (Figures 4A, B). BspPel-th was stable over a broad pH range of 4–11. After incubation at 30°C for 6 h, BspPel-th still showed more than 80% residual enzyme activity (Figures 4C, D). It has been proven that BspPel-th exhibits good stability under alkaline conditions. The residual enzyme activities of BspPel and BspPel-th were 78.5% and 81.8%, respectively, after incubation for 6 h at pH 11. The residual enzyme activity of the recombinant mutant incubated at pH11 was 3.3% higher than that of the wild-type.

**FIGURE 5:**
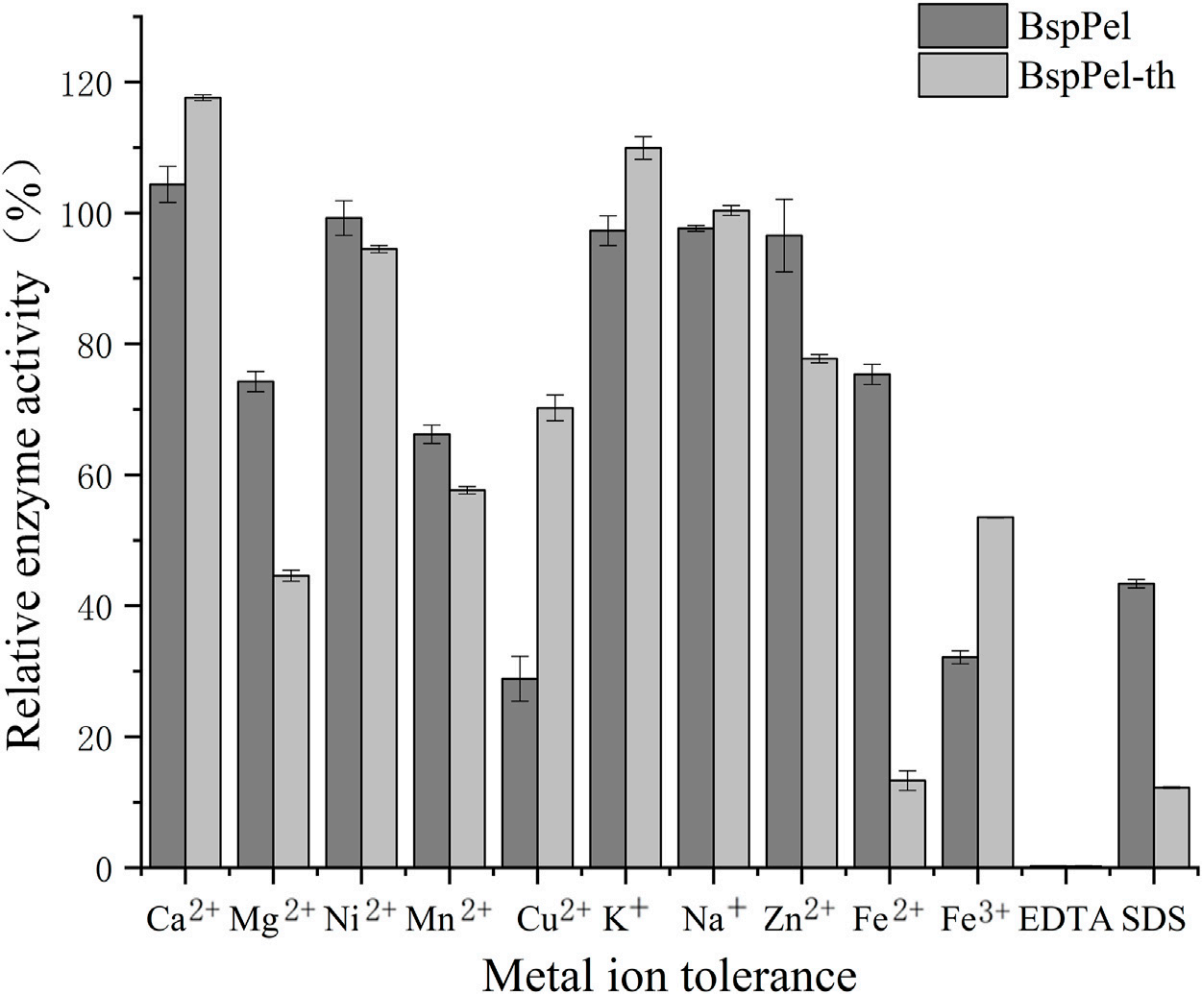
Effects of metal ions on the activity of BspPel and BspPel-th. Values are expressed as the mean of three experiments. Error bars represent standard deviations.

Evaluation of the effects of metal ions and chemicals on the enzyme activities of BspPel and BspPel-th at the respective optimal temperatures revealed that EDTA (0.1%) completely inhibited the activities of both these enzymes. The metallic ions Mg^2+^, Mn^2+^, Fe^2+^ and Fe^3+^ were found to significantly reduce the activity of BspPel-th, whereas no significant effects were detected for other metal ions. Contrastingly, Ca^2+^and K^+^ were observed to enhance the activity of the BspPel-th, and compared with BspPel, we detected an enhancement in the tolerance to Cu^2+^, Na^+^, and Fe^3+^, although a greater sensitive to 0.1% SDS (Figure 5). Most pectin lyase activities of microorganisms depend on Ca^2+^, which is necessary for enzyme binding or salt bridge formation between polygalacturonic acid chains. Therefore, the chelating agent, EDTA, completely inhibited enzyme activity.

### 3.4 Substrate specificity and kinetic parameters

**FIGURE 6:**
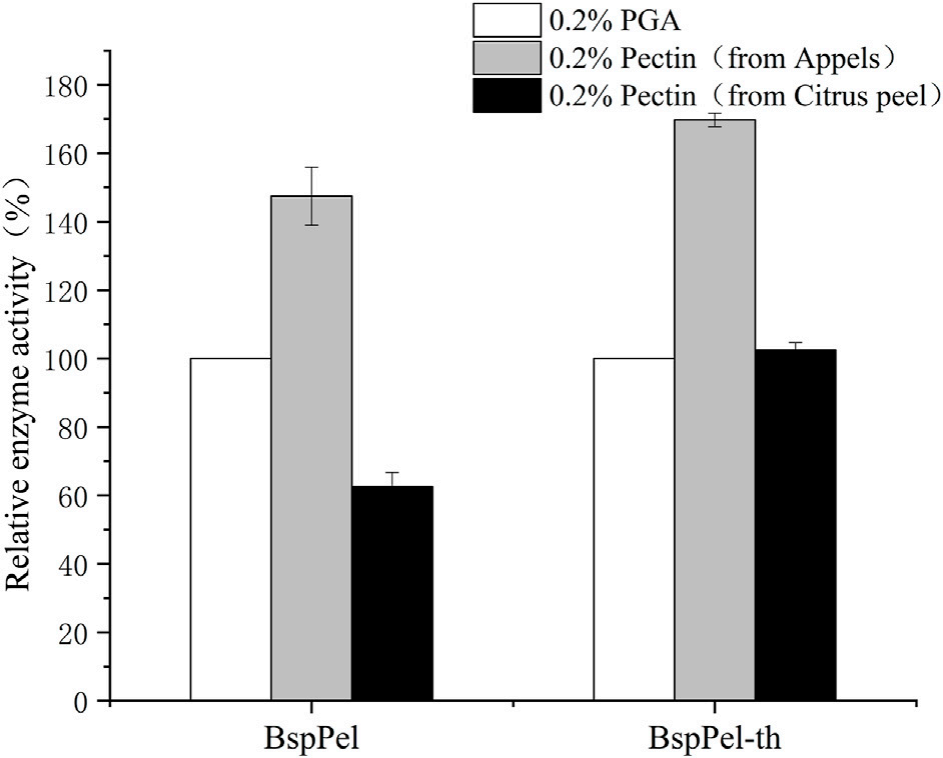
Substrate specificity of BspPel and BspPel-th. Values are expressed as the mean of three experiments. Error bars represent standard deviation.

Substrate specificity of BspPel and BspPel-th was determined with 0.2% (w/v) PGA, pectin (from apples), and pectin (from citrus peel). Compared with PGA (100.0%), BspPel showed 147.45% activity toward pectin (from apples), while only 62.6% activity toward pectin (from citrus peel). The activity of recombinant BspPel-th toward pectin (from apples) and pectin (from citrus peel) was higher than that of BspPel, with 169.75% and 102.45% activities, respectively. It can be seen that recombinant BspPel-th has a better substrate-binding ability than BspPel. Apple pectin has significant substrate affinity. For example, BacPelA shows only 60.7% activity against citrus pectin compared with PGA (100.0%), but 114.7% activity against apple pectin, indicating that pectin (from apples) seems to be the preferred substrate (Figure 6).

**FIGURE 7:**
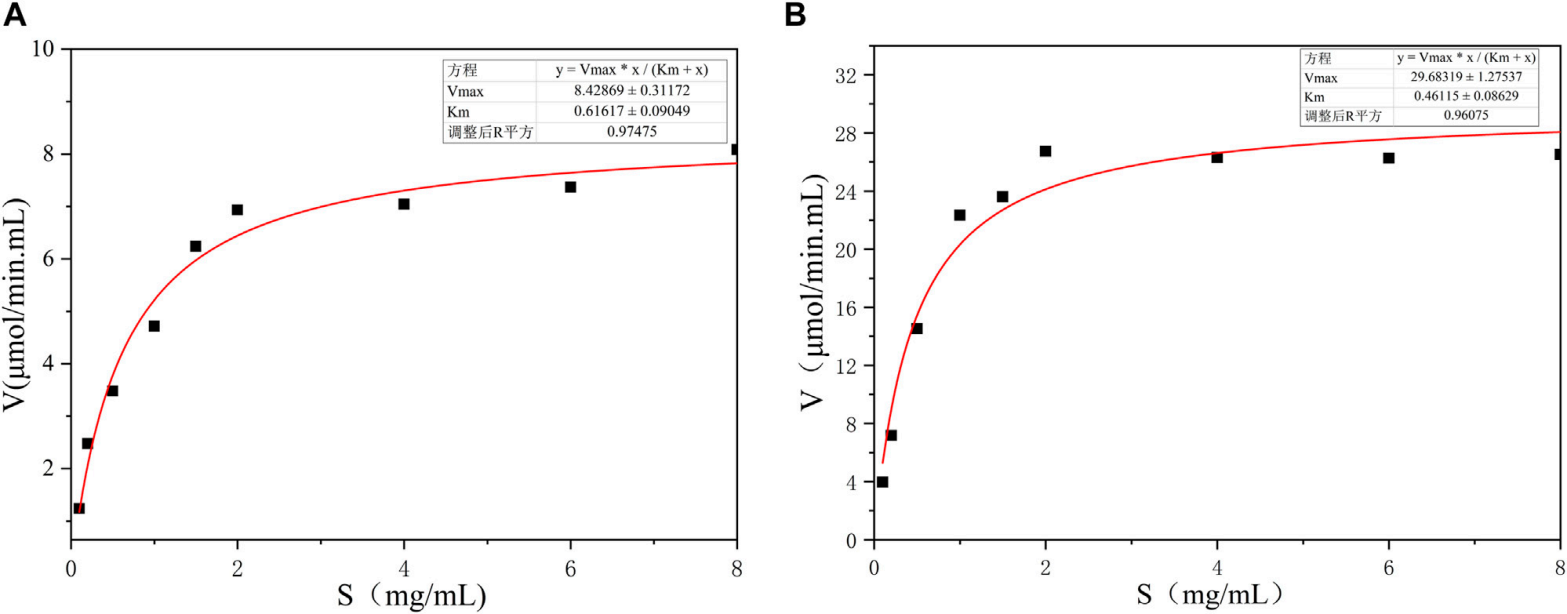
Substrate concentration and velocity curve. **(A)** BspPe, **(B)** BspPel-th.

The kinetic parameters of BspPel and BspPel-th were determined using 0.2% pectin (Appels) as the substrate. The reaction was carried out in 50 mM Gly-NaOH buffer (pH 11.0) at 60°C with pectin concentrations of 0.1–8 mg·mL^−1^. Nonlinear fitting was used to calculate dynamic parameters. *V*_max_ and *K*_*m*_ corresponding to BspPel are 8.43 μmol/min. mL and 0.62 mol·L^−1^, respectively, and the *V*_max_ and *K*_*m*_ corresponding to BspPel-th are 29.6 μmol/min. mL and 0.46 mol·L^−1^, respectively. The smaller the Km value, the greater the affinity for the substrate, which also shows that the affinity between recombinant BspPel-th and the pectin substrate is stronger than that between BspPel (Figure 7).

### 3.5 Three-dimensional structure analysis and dynamic simulation of BspPel-th

The crystal structure of Pel4-N (PDB ID:3VMV) was selected as the template for homology modeling. The sequence similarity was approximately 50%, meeting the homology modeling conditions (sequence similarity>30%). Presently, a large number of pectinases have been characterized based on their biochemical properties, but their crystal structures have not been resolved.

**FIGURE 8:**
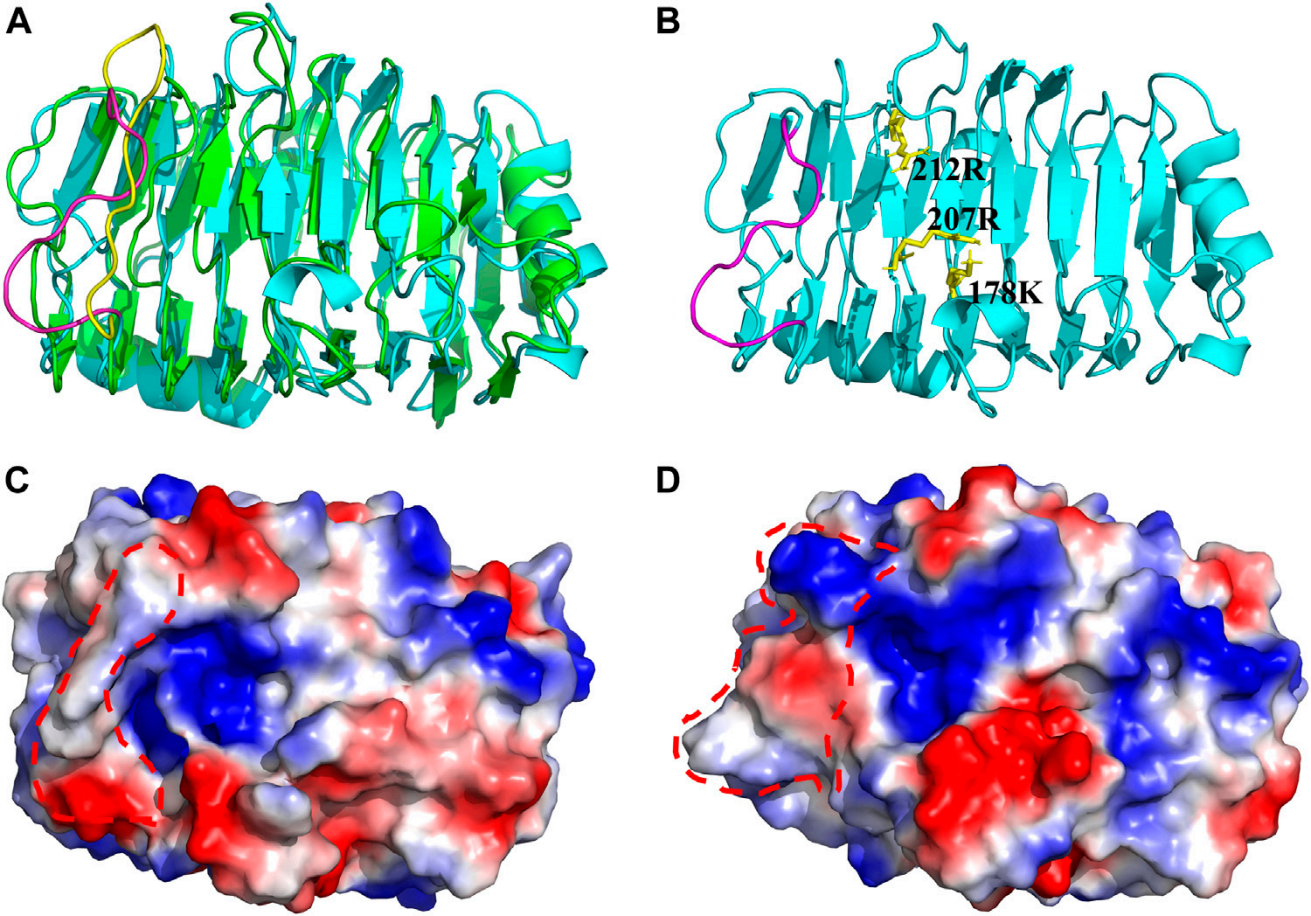
Three-dimensional structure. **(A)** Three-dimensional simulation structure diagram of BspPel and BspPel-th. Yellow represents the amino acid sequence KDPIVSWYSSSP of BspPel loop region, and purple represents the amino acid sequence TNPLGFWHSSRT of BspPel-th loop region, **(B)** The spatial relationship between the active site and the replacement fragment of loop region, **(C)** On the electrostatic potential energy surface of BspPel, the red dotted line indicates the replacement loop position, **(D)** The electrostatic potential energy surface of BspPel-th, the red dotted line indicates the replacement loop position.

The related structure of BspPel-th shows a parallel β-helix topology, where β-sheets fold into a large right-hand coil. The 3D structure prediction results showed that the abrupt loop area was parallel to β-sheets combined with the folded sheet of the spiral structure, and the substrate catalytic pocket was formed (Figure 8). Using the visualization software PyMOL, to display the electrostatic potential energy surface, the replacement loop of BspPel-th and the active center form a larger arginine plane, which can improve the alkali resistance of the enzyme and improve its substrate binding ability.

**FIGURE 9:**
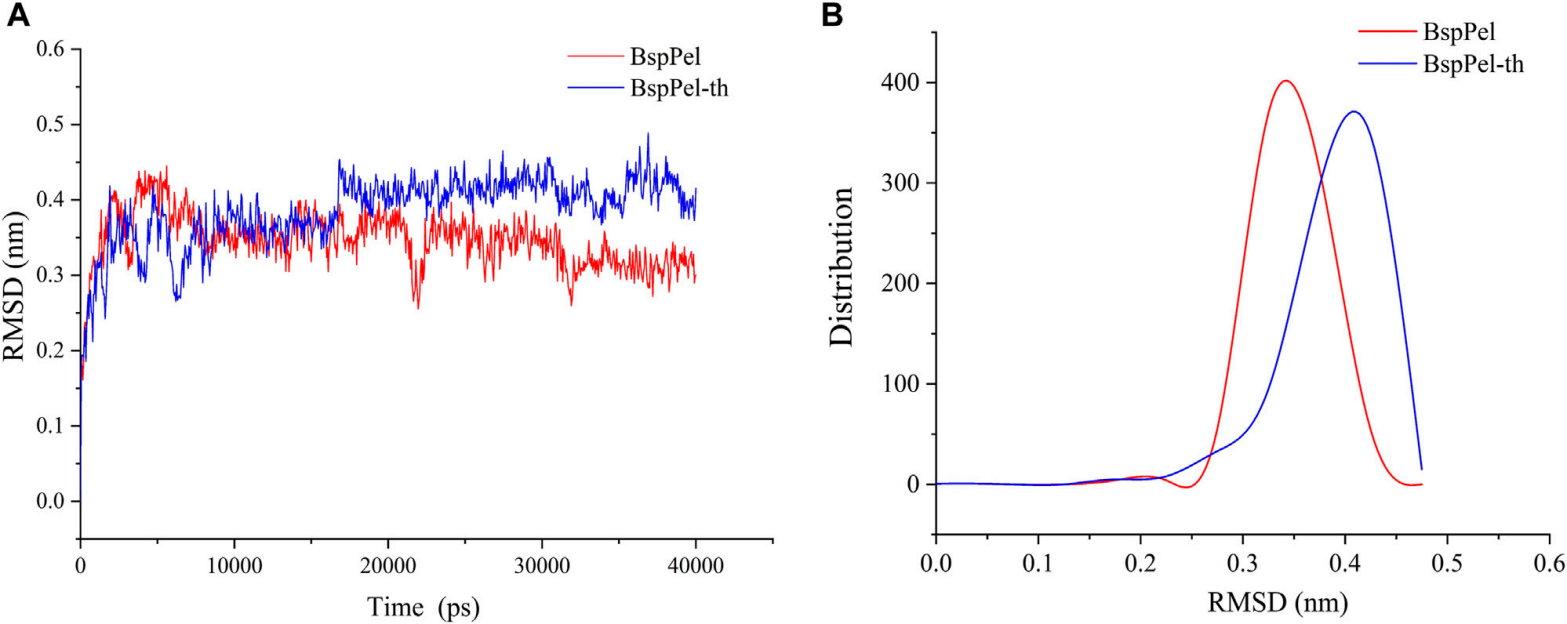
Calculation and distribution of RMSD value. **(A)** The curve of RMSD value of BspPel and BspPel-th after the MD simulation of 40,000 ps at 300 K, **(B)** The distribution of RMSD values of BspPel and BspPel-th respectively.

The root mean square deviation (RMSD) values of BspPel and BspPel-th loop region replacement fragments were calculated after MD simulation, and the simulated trajectories of the two were quite different. The result shows that the RMSD value of BspPel-th after balance is greater than that of BspPel, Simultaneously, the origin software was used to carry out statistical analysis of their RMSD values (Figure 9). The RMSD of BspPel was concentrated at 0.33, whereas the RMSD of BspPel-th was mainly concentrated at 0.43.

### 3.6 Effect of amino acid mutations on alkaline resistance

**FIGURE 10:**
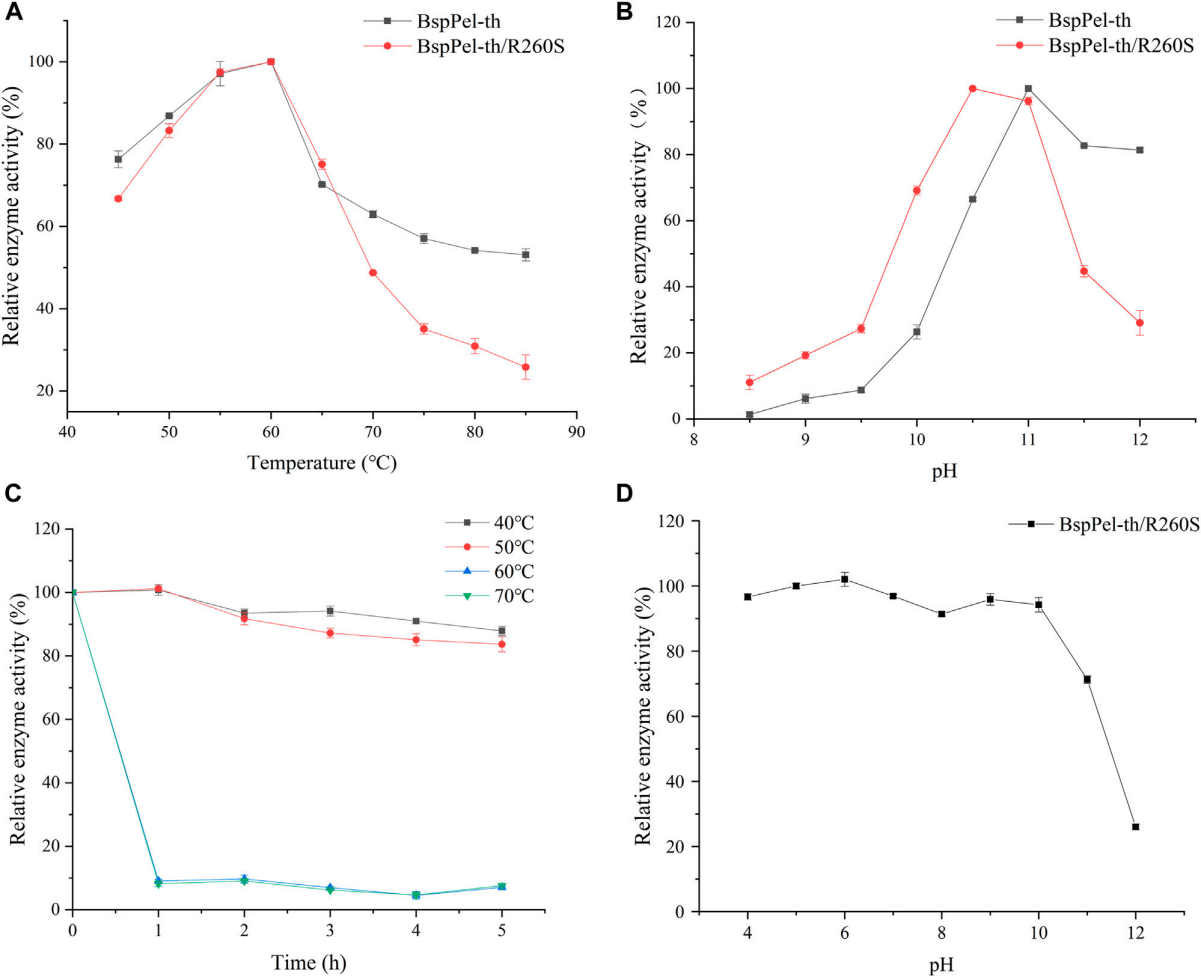
pH and temperature profiles of BspPel-th/R260S. **(A)** optimum temperature, **(B)** optimum pH, **(C)** BspPel-th/R260S temperature stability, **(D)** BspPel-th/R260S pH stability. Values are expressed as the mean of three experiments. Error bars represent standard deviations.

Mutation of arginine at position 260 of BspPel-th to serine at position 260 of BspPel. BspPel-th/R260S was obtained and its enzymatic properties were determined. The results showed that the optimum temperature was consistent with BspPel-th, and the optimum temperature was 60°C. The optimal pH was 0.5 lower than that of BspPel-th, indicating an optimal pH of 10.5, but still 0.5 higher than that of BspPel. It shows good stability at 40°C–50°C and pH stability at 4–10. After incubation under alkaline conditions at pH 11, residual enzyme activity was approximately 70% (Figure 10).

## 4 Discussion

In the papermaking process, an alkaline environment at a moderate temperature is very important for improving fiber quality and effective degumming. Therefore, enzymes with high catalytic activities under alkaline conditions are ideal for the paper industry. BspPel has been reported in previous studies and compared with other reported pectate lyases (Xiao et al., 2012; Yuan et al., 2012; Su et al., 2015), and shows excellent stability characteristics in alkali environments with elevated temperatures. To better adapt the enzyme to the industrial environment, we attempted to improve the enzymatic characteristics of BspPel. Proteins are composed of different amino acids that have unique characteristics and are responsible for folding, function, stability, and enzyme activity. Hydrogen bonds, hydrophobic interactions, ionic bonds, salt bridges, and other factors are essential for promoting protein activity and stability (Wu X. Y. et al., 2020).

This study reports the effect of the loop region of pectin lyase activity on the catalytic efficiency of the enzyme. The loop region is the most flexible part of the enzyme molecule, and the active loop region is involved in the catalytic action of enzyme molecules, usually located at the entrance of the active site, and plays an important role in substrate selectivity, recognition, or promotion of substrate binding. Protein modification of the loop region will also affect the stability and activity of the enzyme (Malabanan et al., 2010). Miao et al. (2021) rationally designed the loop region of a diagonal protease to obtain mutants with improved catalytic efficiency and thermal stability. In this study, we found that rational replacement of the loop region above the active site (AA250-261) resulted in the improvement of BspPel in alkali resistance and enzyme activity.

The surface charge plays an important role in determining the optimal pH of the enzyme. It has been proved that the replacement of lysine with arginine can improve the alkali resistance of some enzymes (Miao et al., 2021). There are two types of charged amino acids in the loop region of BspPel: one acidic amino acid (D251) and one basic amino acid (K250). Furthermore, analysis of the structure of alkaline cellulase K revealed that the relative number of Arg, His, and Gln residues in the protein increased compared to that of non-alkaline cellulases, while the number of Asp and Lys residues decreased (Deng et al., 2014). Therefore, Adjusting the distribution of amino acids on the protein surface is a common method of improving protein alkali resistance. After fragment substitution, the optimal pH after mutating arginine to serine decreased. This may be due to the fact that R260 in the loop region is able to form a larger arginine plane with the active site, and one side chain of arginine has a guanidine group at one end, favoring the formation of multiple hydrogen bonds. Alkaline amino acids easily form hydrogen bonds with the surrounding hydrophilic amino acids or water molecules, forming a relatively stable shell for protein molecules (Shirai et al., 2001). In this study, the replacement loop of BspPel-th contains two basic amino acids, R260 and H257, this may be one of the reasons for significant improvement in alkali resistance.

Molecular dynamics simulation is the most commonly used method for analyzing protein flexibility. Molecular dynamics simulation analysis of proteins at different temperatures is of great significance for protein modification (Li et al., 2019). The simulation results show that the mutated loop region has higher flexibility, which effectively increases the flexibility of the substrate-binding pocket, and the catalytic active site makes it easier to contact the substrate, thus improving catalytic efficiency. We used molecular dynamics simulation combined with SWISS-MODEL simulation to generate the three-dimensional structure of BspPel-th, but protein crystal structures with high complexity, this may affect the evaluation of enzyme molecular functional characteristics, and X-crystal diffraction and other methods can be used to analyze more accurate crystal structures in the future, providing a strong basis for the analysis of enzyme molecular spatial structure.

## 5 Conclusion

In this study, we enhanced the activity of the alkaliphilic pectate lyase BspPel by replacing 12 amino acids in its loop region (AA250-261) with the corresponding 12 amino acids from the highly alkaliphilic pel4-N. The optimal pH of the obtained BspPel-th was significantly higher than that of the wild type. The optimum pH of BspPel-th was 11, which was higher than the optimum pH of BspPel. The specific enzyme activity of BspPel-th was four times that of the wild type enzyme. Moreover, it exhibits good alkali and heat resistance characteristics. The resulting BspPel-th is a good candidate for strong alkali industrial processes. This study provides an effective strategy to improve the alkaline tolerance of other enzymes, whose intrinsic properties hinder their broader applications.

## Data Availability

The datasets presented in this study can be found in online repositories. The names of the repository/repositories and accession number(s) can be found in the article/Supplementary material.
